# Empowering indigenous Papuan fishermen based on local potential

**DOI:** 10.3389/fsoc.2026.1742705

**Published:** 2026-02-03

**Authors:** Matheys Sibi, Renida J. Torobi, Usman Idris

**Affiliations:** 1 Doctoral Program in Social Sciences, Postgraduate Program, Cenderawasih University, Jayapura, Indonesia; 2 Government Science Study Program, Faculty of Social and Political Sciences, Cenderawasih University, Jayapura, Indonesia; 3 Faculty of Social and Political Sciences, Anthropology Department, Cenderawasih University, Jayapura, Indonesia; 4 Doctoral Program in Social Sciences, Faculty of Social and Political Sciences, Cenderawasih University, Jayapura, Indonesia

**Keywords:** empowerment, fishermen, indigenous Papuans, Indonesia, local potential

## 1 Introduction

The problem of structural poverty on fishermen is a major concern for the Ministry of Maritime Affairs and Fisheries of Republic Indonesia (Gunawan et al., 2023). However, this problem is often only viewed superficially by policy makers. Revealing the truth is not easy, because cultural barriers hinder the understanding of policy makers and the context that exists in society. Moreover, the ethnic diversity in the archipelago is very high, which further complicates the understanding process. As a result, most development programs in coastal areas have not been running well. The resources obtained from these programs do not empower the fishermen who work, but become assets for capital owners and business owners (Pomeroy et al., 2020).

In the context of indigenous Papuan fishermen, the situation is quite chronic when viewed from the perspective of welfare in carrying out their livelihoods. This is understandable, given the massive process of exploration and exploitation of fishery resources as part of global capitalism. Indigenous Papuan fishermen still apply patterns of resource utilization and management that are subsistence-based amid limited fishing equipment on the one hand, but on the other hand, they uphold the values of harmony with nature and the environment so that the utilization is not reckless but managed wisely to meet their needs (Kadir et al., 2021; Idris et al., 2021; Hijjang et al., 2018). As a result, there is a gap between migrant fishermen groups originating from Bugis-Makassar, Buton, Maluku, Kei, Java, Madura, and other groups, and local fishermen who are mostly indigenous Papuans (Lampe, 2012; Danugroho et al., 2025; Idris et al., 2024; Setiadi and Sumini, 2023).

To address this issue, various policies have been formulated to empower indigenous Papuan fishers. However, ironically, they still face various structural and implementation barriers. One root of the problem is a development approach that tends to be formalistic, project-oriented, and lacking community participation. National-scale programs such as the Coastal Community Development Project (CCDP) and the *PNPM Mandiri Kelautan dan Perikanan* (National Program for Maritime Affairs and Fisheries) often emphasize providing physical assistance or short-term training, without ongoing, contextualized support for local cultural values and institutions (Tjilen et al., 2018; Béné and Neiland, 2004). In the implementation of the CCDP program in Merauke, Papua, fishing gear assistance provided included a 40-horsepower motorboat and simple fishing gear such as nets and fishing rods. However, this assistance was not in line with the habits of indigenous Papuan Fishermen, who still use traditional technology and uphold environmental harmony values. Consequently, the equipment was rarely used and eventually abandoned (Tjilen et al., 2018). Technically, the assisted boats capsized easily because they were not designed to withstand the large waves in the open sea of Merauke, posing a high risk to fishermen. Furthermore, the assistance did not include skills training in operation and maintenance, making it difficult for fishermen to utilize them optimally.

The failure of the CCDP program was not only due to a top-down approach, but also to mistargeted beneficiaries, forms of assistance that were incompatible with local culture and practices, and unsustainable funding allocation. This highlights the need to analyze empowerment as a policy concept, not simply as technical assistance. Therefore, Carrick-Hagenbarth (2021) stated that many community-driven initiatives fail because they fail to shift real power or control to the community as the subject of development, remaining merely administrative and ceremonial. This results in local communities remaining marginalized in decision-making, while increasing dependence on external actors.

In the Papuan context, the renewal of Special Autonomy through Law No. 2 of 2021 should open up significant opportunities for more meaningful empowerment of the traditional fisheries sector. However, to date, there are no technical regulations specifically governing the involvement and protection of the rights of indigenous Papuan fishers within the framework of the new Special Autonomy (Otsus) implementation. The absence of derivative regulations poses a serious obstacle to realizing resource management based on local wisdom (Hijjang et al., 2018; Kadir et al., 2021). In fact, a co-management approach based on customary institutions could be a solution to address the unequal power relations between local fishers and modern capitalists. In the absence of implementing regulations that bridge Papua's uniqueness in coastal management, a uniform national policy actually exacerbates inconsistencies and reinforces the structural marginalization of indigenous fishing communities (Indrawan et al., 2019; Visser, 2001). This contrasts even more sharply with the trade sector in Jayapura City, Papua Province, which already has Regional Regulation Number 10 of 2018 concerning the Protection and Empowerment of Local Traders or Indigenous Papuans (OAP). The regulation explicitly stipulates structural affirmative action for indigenous fishermen, including priority access to business locations, business capital protection, and ongoing development by local governments (Idris et al., 2023). Thus, a comparison with the trade sector underscores the urgency of similar affirmative action regulations in the fisheries sector to promote social justice and strengthen the capacity of indigenous fishermen.

The main objective of this paper is to formulate a contextual empowerment strategy for indigenous Papuan fishermen, emphasizing mentality transformation, strengthening local institutions, and recognizing local wisdom in coastal resource management. This paper stems from concerns about structural marginalization and the failure of top-down, non-participatory development programs, and aims to propose an alternative approach that positions fishing communities as the primary subjects of development through participatory dialogue, ongoing mentoring, and the development of sustainable local potential.

## 2 Discussion

The concept of “empowerment” in the context of Papuan indigenous fishers must be understood as a process of transforming power relations that positions communities as the primary actors in resource management, not simply increasing technical capacity or providing economic assistance (Béné and Neiland, 2004). Empowerment truly encompasses legal and institutional recognition of fishers' rights and sovereignty (co-management, customary institutions), strengthening their economic position through market access and equitable distribution, and strengthening local knowledge and participatory social organizations. In the Papuan context, recognizing local systems such as sasi laut (sea stipulations) or puyakhabhu (sea stipulations) is key to creating equitable and sustainable development. Unfortunately, a top-down approach remains dominant and often neglects inclusive institutionalization dialogue and substantive changes in power structures.

The strategies that need to be developed to empower fishing communities to improve their quality of life are, *first*, development programs that emphasize a revolution in mentality, not just physical empowerment programs, and second, participation contributed by the community only as development implementers, which shows that they are actually powerless but dependent on the development program (Indrawan et al., 2019; Sitorusof, 2017; Visser, 2001). Programs that target the mentality of fishermen should not be mere discourse or temporary activities that are merely ceremonial euphoria, but must be followed by a periodic and continuous mentoring process for the institutionalization and internalization of attitudinal changes in the structure of the community's mentality.

Mentality is a very important and vital aspect in constructing the direction of community development (Kaptaner, 2023; Koentjaraningrat., 1988). If the local community always has a mentality of defeatism, of dependence on affirmative policies, then it is certain that they will remain stuck in place, unable to move forward at all. Building a “fighting” mentality in the context of becoming a powerful subject to determine the direction of development in one's own village is important in shaping the character of the local community. Strengthen the mentality by instilling a strong understanding and desire to change, to be able to accelerate with ongoing progress in order to keep pace with the quality of migrant human resources. Otherwise, Papuans will forever remain spectators in their own homes. It is time to take action and strengthen individual awareness to change by forming a mentality that does not depend on affirmative policies and all kinds of assistance that actually pamper and make Papuans lazy. The spirit of work in building the ethos and giving birth to the subjectivity of the Papuan people, especially fishermen, to empower themselves, their families, their relatives, their clans, their communities, and the Papuan people in general for a more humane mutual progress and to realize a better standard of living.

Empowering indigenous Papuan fishers requires a mental revolution approach aligned with structural and institutional change. Transformations in perspective, work ethic, and independence must be accompanied by formal recognition of local knowledge and customary institutions and village deliberations as the basis for resource management. Policies must guarantee community control over resources, active participation in planning, and support for sustainable financing schemes that strengthen local fiscal independence. Without this, mentality change will be mere rhetoric without real impact. Successful development in Papua depends on local strengths, not a centrally-driven model that ignores the customary context.

As a complement to the mentality and institutional approaches, it is also important to critically examine the policy dimensions that contribute to the failure of empowerment, for example in the implementation of programs like the CCDP, so that the analysis does not stop at merely normative aspects. The failure of empowerment policies like the CCDP can be examined from three main dimensions that are often overlooked in policy analysis: first, errors in targeting beneficiaries that do not take into account the social and cultural structures of indigenous Papuans. The CCDP program in Merauke actually benefits migrant groups like the Bugis-Makassarese more than lndigenous Papuan fishermen, who rely on subsistence. This inequality arises because migrant fishermen have better technology and access to capital, preventing indigenous groups from competing fairly for assistance. second, forms of assistance that do not match real needs and local fishing practices such as the use of traditional technology; and third, allocation of funds that are more oriented toward short-term projects without ongoing mentoring or training schemes. The CCDP program in Merauke was implemented only during the physical assistance phase, such as providing motorboats and fishing gear, without ongoing mentoring or follow-up programs after distribution. For example, fishermen did not receive regular training on how to use and maintain the equipment, resulting in it going unused and ultimately being abandoned.The CCDP program in Merauke lasted less than 12 months (Tjilen et al., 2018; Irawan and Tanzil, 2020). Yet, achieving structural empowerment (such as mentality change, institutional transformation, and technological adaptation) requires consistent medium- to long-term interventions of at least 2–5 years (Carrick-Hagenbarth, 2021). These three aspects demonstrate that empowerment cannot be reduced to the mere distribution of tools or short-term training, but must be accompanied by context-based policy engineering and active local participation.

*Second*, capacity building in line with local potential. The local potential possessed by local communities, such as that of indigenous Papuan fishermen, is a cultural asset that can be used to enter the struggle and arena of contestation for capital resources that is currently underway (Paulangan et al., 2020; Indrawan et al., 2019). The local competence referred to here is not an entity separate from the identity and environment of the local community, but rather something that is integrated with each other. This includes traditional knowledge, special skills formed from experience, patterns of environmental utilization and management, unique cultural characteristics, and physical, social, and cultural advantages that are attractive and beneficial. These local capacities only need to be refined and/or contextualized with a development perspective for empowerment and improvement of the community's standard of living. Of course, not all of these local potentials are relevant and contextual for the purposes of sustainable development.

However, this intersection is necessary to harmonize what is needed in the development implementation process. The basis for determining development and empowerment programs is rooted in local potential that can be developed and is in line with sustainable development goals. However, what needs to be underlined here is the possibility of opening an emancipatory and participatory dialogue between the formulators, facilitators, and the community itself in the process of planning, implementing, and evaluating development and/or empowerment programs that are carried out to accommodate the aspirations of the community and the capabilities of the facilitators so that the policies appear realistic to be articulated into a concrete program.

*Third*, continuous assistance toward community independence and innovation. Until now, development projects for community empowerment have failed because the implementation stage has seemed merely formal (Carrick-Hagenbarth, 2021; Tjilen et al., 2018; Béné and Neiland, 2004; Visser, 2001). This means that programs are carried out and then abandoned, with no continuous and sustainable assistance. So, it seems that the only thing that matters is reporting on paper, along with documentation, proposals, and budgeting. However, this does not lead to measurable success. In fact, to run a development program, participation and the transfer of knowledge in the context of externalizing and enculturating the empowerment program so that it permeates the mentality and subconscious of the community, which is both the subject and object of development, requires assistance in the form of continuous socialization, demonstration, and objectification to shape understanding from past experiences.

Community involvement and the instructional process of direct action with mentoring leave a meaningful impression, and if done continuously, will gradually become a new habit and shape internalized understanding and behavior so that it is understood as a reality that has become a reality. In line with this, the changes that occur and are carried out continuously will gradually be internalized, thereby shaping mentalities, increasing participation, and empowering the community in their choices and orientation (in life) to improve their standard of living.

## 3 Conclusion

The empowerment of indigenous Papuan fishermen must be based on a comprehensive and contextual approach that emphasizes not only physical assistance but also mental transformation, institutional strengthening, and the recognition and integration of local wisdom into coastal resource governance. Structural barriers, a top-down development approach, and the absence of technical regulations protecting the rights of indigenous fishermen indicate that current development efforts have not addressed the root of the problem. The proposed strategy includes a mental revolution in communities to reduce dependence on aid, capacity building based on local potential, and ongoing mentoring that encourages community independence and innovation. Therefore, true empowerment requires a commitment to developing participatory, equitable, and sustainable policies that prioritize the active role of local communities as the primary subjects of development.
